# Disruption of Synaptic Vesicle Trafficking in Alzheimer’s and Parkinson’s Disease: Mechanisms and Therapeutic Implication

**DOI:** 10.3390/ijms27073089

**Published:** 2026-03-28

**Authors:** Youyang Zhu, Lianna Zhao, Yingming Li, Miao Tian, Yingdi Liao, Jinqing Huang, Peixin Guo, Yuhuan Xie

**Affiliations:** 1 First Clinical Medical College, Yunnan University of Chinese Medicine, Kunming 650500, China; zyy980213@163.com (Y.Z.); 18332791312@163.com (M.T.); ying19961215@126.com (Y.L.); 2 College of Traditional Chinese Medicine, Yunnan University of Chinese Medicine, Kunming 650500, China; cjs48ws@163.com (L.Z.); lym_222@163.com (Y.L.); 15677588716@163.com (J.H.); 3 The College Based Key Laboratory of Yunnan in Aromatic Chinese Herbal Research, Kunming 650500, China; 4 College of Ethnic Medicine, Yunnan University of Chinese Medicine, Kunming 650500, China; 5 Science and Technology Department, Yunnan University of Chinese Medicine, Kunming 650500, China

**Keywords:** synaptic vesicle trafficking, Alzheimer’s disease, Parkinson’s disease, SNARE complex, protein aggregation

## Abstract

Alzheimer’s (AD) and Parkinson’s disease (PD) are prominent neurodegenerative disorders characterized by early synaptic loss, which correlates more closely with clinical symptoms than neuronal death. This synaptic impairment is primarily driven by disruptions in synaptic vesicle (SV) trafficking, a critical process for maintaining synaptic integrity through a tightly regulated cycle involving clustering, docking-priming, Ca^2+^-triggered fusion, and endocytosis. In AD, amyloid-β (Aβ) oligomers interfere with SNARE-mediated fusion and endocytosis, while hyperphosphorylated tau obstructs vesicle mobility and docking, resulting in cumulative toxicity that aggravates SV defects. Conversely, in PD, α-synuclein (α-syn) aggregation alters vesicle clustering, membrane fusion, and recycling, and these effects are further influenced by Leucine-rich repeat kinase 2 (LRRK2)-Rab-related trafficking defects and the selective vulnerability of dopaminergic terminals. Different from previous reviews that address synaptic dysfunction in a broader manner, the present review is specifically organized around the SV trafficking cycle and compares both shared presynaptic endpoints and disease-specific upstream mechanisms in AD and PD. In addition, recent mechanism-oriented therapeutic strategies are summarized. This vesicle-cycle-centered perspective may provide a clearer framework for understanding presynaptic pathology and for guiding the development of earlier and more targeted interventions.

## 1. Introduction

Synaptic transmission serves as the fundamental mechanism of inter-neuronal communication, enabling the central nervous system to interpret and respond to internal and external stimuli. This process underpins complex physiological functions, encompassing sensory, motor, cognitive, and emotional processing. Within this intricate communication network, synaptic vesicle (SV) trafficking plays a pivotal role in neurotransmitter release at presynaptic terminals.

The historical foundations of our understanding of SV dynamics can be traced to the seminal work of Bernard Katz in the mid-20th century. Utilizing electron microscopy, he postulated the hypothesis of quantal neurotransmitter release, thereby establishing the cellular basis for neural information transmission [1]. SV trafficking has long been the subject of extensive scientific investigation. The proper cycling of SVs is vital for preserving synaptic function. The SV cycling process includes vesicle biogenesis, axonal transport, docking and priming at active zones, calcium-triggered membrane fusion, and subsequent compensatory endocytosis [2,3,4]. It is not only responsible for neurotransmitter transport and signal transduction, but it is also closely linked to factors such as energy metabolism and protein homeostasis. As SV cycling is critical for sustaining inter-neuronal communication, dysregulation at any stage—such as impaired assembly of the soluble N-ethylmaleimide-sensitive-factor attachment protein receptor (SNARE) complex or delayed clathrin-mediated endocytosis (CME)—can induce synaptic dysfunction and ultimately result in synaptic loss [5]. Such synaptic loss has been identified as a critical early event in neurodegenerative diseases.

Neurodegenerative diseases (NDs), such as Alzheimer’s disease (AD) and Parkinson’s disease (PD), have emerged as an increasingly severe medical burden in aging populations [6,7]. While these disorders are characterized by distinct clinical features and target different brain regions, they share a fundamental pathological hallmark: synaptic loss [8]. Historically, research has concentrated on end-stage pathological changes that occur following neuronal death. However, accumulating evidence suggests that the onset of cognitive decline and motor impairment is more closely associated with reduced synaptic density than with neuronal loss [9,10]. Furthermore, recent findings propose that hallmark pathogenic proteins in NDs may modulate presynaptic function under physiological conditions [11,12]. This indicates that synaptic dysfunction in the context of disease may not only result from the physical obstruction caused by aggregated pathogenic proteins but also from the disruption of their physiological roles and pathological interactions among these proteins that hinder SV trafficking. For instance, while α-synuclein (α-syn), a protein associated with PD, normally functions as a chaperone facilitating SNARE complex assembly and vesicle fusion, its pathological overexpression has been shown to suppress vesicle mobility [13,14]. Similarly, tau protein, traditionally regarded as a stabilizer of microtubules, has been identified at presynaptic sites where it binds to SVs under pathological conditions, consequently impeding their mobilization [15].

Although synaptic dysfunction has been widely discussed in AD and PD, fewer reviews have examined these disorders specifically through the framework of the SV trafficking cycle. The present review focuses on how disease-related proteins disrupt distinct steps of this cycle, including vesicle clustering, docking and priming, SNARE-dependent fusion, and endocytosis. Our goal is not only to summarize known presynaptic changes, but also to compare shared mechanisms of synaptic failure with disease-specific upstream triggers. In this regard, AD is discussed mainly as a disorder in which amyloid β (Aβ) and tau converge on vesicle trafficking through partly indirect and cooperative mechanisms, whereas PD is discussed as a disorder in which α-syn and Leucine-rich repeat kinase 2 (LRRK2)-related pathways act more directly on presynaptic vesicle organization and recycling. By organizing the literature around the vesicle cycle itself, this review aims to provide a more integrated view of how similar presynaptic endpoints arise from different molecular insults, and why these distinctions may matter for mechanism-based therapy.

## 2. The Physiological Mechanisms of SV Recycling

### 2.1. Vesicle Clustering and Pool Maintenance

Under resting conditions, the presynaptic terminal contains hundreds to thousands of SVs, the majority of which do not directly participate in release but instead form a reserve pool [16]. Traditionally, synapsins have been thought to restrict vesicle diffusion by crosslinking SVs to the actin cytoskeleton [17,18]. In recent years, groundbreaking studies have introduced the concept of liquid–liquid phase separation (LLPS) [19,20,21]. Milovanovic and colleagues demonstrated that synapsin I, through its intrinsically disordered region (IDR), can undergo phase separation within SV-rich domains, forming droplet-like condensates [22]. This mechanism not only maintains the clustering and recycling of the SV pool, enabling rapid mobilization when needed, but also preserves the stability of key SV trafficking proteins such as presynaptic Rab-3-interacting molecule (RIM) 1/2 and Munc13 [23]. Disruption of this dynamic balance may lead to aberrant protein aggregation.

### 2.2. Docking, Priming, and SNARE Assembly

Before the arrival of an action potential, a subset of SVs is recruited to the active zone membrane, undergoing docking and priming to become part of the readily releasable pool (RRP) [24,25]. This process is primarily orchestrated by highly conserved active zone proteins, including RIM, Munc13, and Rab GTPases. At its core lies the assembly of the SNARE complex: the vesicle membrane-associated SNARE, predominantly synaptobrevin 2/Vesicle-associated membrane protein 2 (VAMP2), recognizes and pairs with plasma membrane-associated SNAREs, syntaxin 1 and Synaptosomal-associated protein 25 (SNAP 25) [16,26,27]. Munc18 and Munc13 function as essential chaperones in this process—Munc18 binds to and stabilizes the closed conformation of syntaxin 1, whereas Munc13 promotes its opening, exposing the SNARE domain to initiate assembly of the four-helix bundle [28,29,30]. This “half-zippered” SNARE complex then awaits action potential-driven depolarization and calcium influx as the final trigger for efficient neurotransmitter release.

### 2.3. Calcium-Triggered Fusion

Upon action potential-induced depolarization, voltage-gated calcium channels open, allowing Ca^2+^ influx that produces transient nanodomain hotspots. At this point, the vesicular calcium sensor synaptotagmin 1 (Syt1) acts in a dual capacity as both a “brake” and a “trigger” [31]. Under low-calcium conditions, Syt1 may cooperate with complexin to clamp the SNARE complex and prevent spontaneous vesicle fusion [32]. In contrast, at elevated calcium levels, the C2 domains of Syt1 bind to Ca^2+^ and insert into the phospholipid bilayer of the plasma membrane, inducing membrane curvature and simultaneously releasing the clamp on the SNARE complex [33,34]. This transition allows the “half-zippered” SNARE complex to undergo rapid full zippering, forcibly opposing the vesicle and plasma membranes, thereby opening the fusion pore and triggering neurotransmitter release [35].

### 2.4. Endocytosis and Recycling

To maintain plasma membrane area homeostasis and replenish the vesicle pool, vesicular membranes must be retrieved following fusion. For decades, CME has been regarded as the predominant mechanism for SV recycling [36,37,38,39]. The frequency and kinetics of CME alone, however, are difficult to reconcile with the sustained demands of high-frequency neuronal activity in vivo. Advances in experimental methodology have revealed additional pathways: using optogenetics combined with “flash and freeze” electron microscopy, Watanabe and Jorgensen’s group identified the existence of ultrafast endocytosis [40,41]. More recent work employing millisecond-scale, time-resolved in situ cryo-electron microscopy has further elucidated the biophysical sequence linking SV release to rapid retrieval—termed “kiss shrink escape/fusion”. In this process, a vesicle forms a nanometer-scale fusion pore with the presynaptic membrane (“kiss”), rapidly constricts into a smaller vesicle with nearly half the original surface area (“shrink”), and ultimately undergoes retrieval predominantly via an “escape” route, with a minority proceeding to full fusion [42].

### 2.5. Physiological Functions of Synapse-Related Proteins in SV Trafficking

Efficient synaptic transmission depends on the coordinated action of synapse-related proteins across the SV trafficking process. At resting terminals, synapsins help retain vesicles within the reserve pool, while liquid–liquid phase separation further supports vesicle clustering and the local organization of presynaptic proteins [17,18,20]. As vesicles are recruited to the active zone, VAMP2, syntaxin-1, and SNAP-25 form the core SNARE machinery, and this process is shaped by key regulatory proteins such as Munc13 and Munc18, which control docking, priming, and release readiness [29,30]. Fusion is then triggered by Ca^2+^ influx through the action of synaptotagmin-1, which couples calcium sensing to rapid membrane merging [31,32]. After release, vesicle membranes are retrieved and recycled through endocytic pathways involving clathrin, AP2, endophilin, dynamin, and synaptojanin 1 [36,37]. These proteins do not act in isolation; rather, they form a functionally connected system in which disturbance at one step can impair the entire vesicle cycle. This framework is important for the present review because disease-related proteins in AD and PD do not cause nonspecific synaptic injury alone, but instead disrupt defined presynaptic proteins and pathways, leading to selective defects in vesicle clustering, fusion, or recycling.

**Figure 1:**
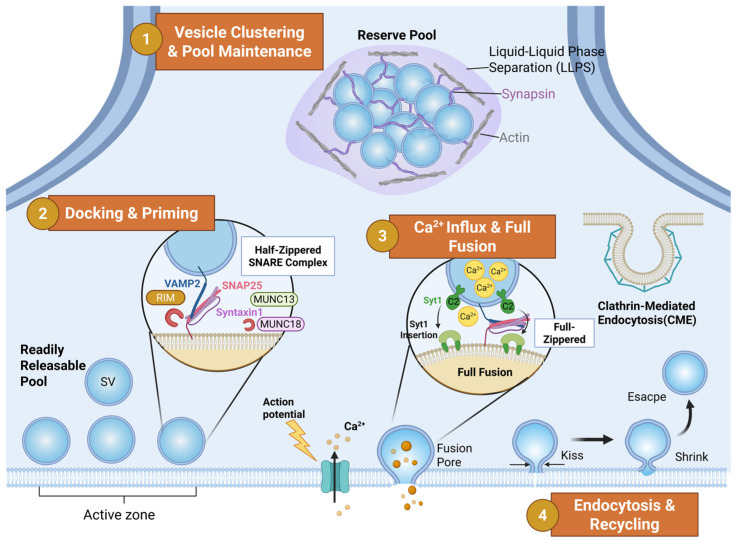
The physiological mechanisms of the SV trafficking cycle at the presynaptic terminal. ① Vesicle Clustering and Pool Maintenance: Under resting conditions, Synapsin I undergoes liquid–liquid phase separation (LLPS), forming droplet-like condensates that cluster SVs into a reserve pool, thereby restricting their diffusion. ② Docking, Priming, and SNARE Assembly: SVs are recruited to the active zone membrane. Orchestrated by scaffold proteins RIM, Munc13, and Munc18, the vesicular v-SNARE (VAMP2) and plasma membrane t-SNAREs (Syntaxin-1/SNAP-25) assemble into a “half-zippered” state, preparing the vesicle for rapid release. ③ Calcium-Triggered Fusion: Upon action potential arrival, voltage-gated calcium channels open, allowing Ca^2+^ influx. Ca^2+^ binds to the C2 domains of Synaptotagmin-1 (Syt1), triggering its insertion into the plasma membrane. This action catalyzes the full zippering of the SNARE complex (“Full-zippered” state), forcing the fusion pore open for neurotransmitter release. ④ Endocytosis and Recycling: Following fusion, vesicle membranes are retrieved. The diagram depicts the ultrafast “Kiss-Shrink-Escape” pathway, where vesicles form a transient pore, shrink in surface area, and rapidly detach. The traditional Clathrin-mediated endocytosis (CME) pathway (coated pit) is shown as an alternative mechanism.

SVs are essential mediators of information transfer within the nervous system, ferrying neurotransmitters from the neuronal interior to the active zones of the presynaptic membrane under the influence of the aforementioned synaptic proteins. Following docking and fusion, neurotransmitters are released into the synaptic cleft, where they bind to receptors on the postsynaptic membrane to complete signal transmission. After release, SVs are retrieved via endocytosis at the presynaptic membrane, initiating a new cycle of vesicle trafficking (Figure 1). Beyond its role in neurotransmitter transport, SV trafficking participates in the movement of diverse pathological products. Consequently, the impairment of vesicle trafficking can disrupt neurotransmitter delivery and compromise the clearance of pathological cargo, thereby accelerating synaptic loss and ultimately contributing to neuronal death.

## 3. SV Trafficking and Neurodegenerative Diseases

### 3.1. Alzheimer’s Disease

Alzheimer’s disease (AD) is classically characterized by two hallmark pathological features: the deposition of extracellular Aβ plaques and the intracellular accumulation of neurofibrillary tangles (NFTs) caused by the hyperphosphorylation of tau protein [43,44]. In the early stages of cognitive decline, however, synaptic dysfunction and the consequent loss of synapses represent a critical pathogenic event [9]. Emerging evidence over recent years suggests that AD is a synaptopathy in which soluble Aβ oligomers and presynaptic tau protein act in concert to severely disrupt SV trafficking, in essence.

Current evidence indicates that pathological products in AD can impair vesicle fusion, release, and trafficking, thereby contributing to synaptic dysfunction [45,46]. Compared with healthy individuals, the levels of SNARE complexes—critical drivers of vesicle fusion—are markedly reduced in the presynaptic active zones of the AD brain [47]. Consistent with human findings, the brains of APP/PS1 transgenic mice exhibit significantly lower SNARE complex levels than wild-type controls. Experimental data demonstrate that Aβ_1–42_ oligomers can bind the SNARE protein syntaxin 1A with high affinity, thereby blocking assembly of the SNARE complex and preventing SNARE-mediated fusion pore formation [48]. This inhibition reduces vesicle fusion with the presynaptic membrane and attenuates neurotransmitter release. Live cell imaging of SV trafficking in embryonic-day-18 hippocampal neurons from Sprague Dawley rats revealed that Aβ_1–42_ can induce the phosphorylation of Ca^2+^/calmodulin-dependent protein kinase IV (CaMKIV) and synapsin. The underlying mechanism likely involves elevated intracellular Ca^2+^ levels, which profoundly suppress axonal SV trafficking [49].

Beyond the impact of Aβ deposition on SVs, the role of tau protein has attracted increasing attention in recent years. Traditionally, tau has been regarded as a microtubule-associated protein that stabilizes neuronal microtubules, thereby promoting axonal growth [12]. However, high-resolution imaging over the past decade has revealed that tau is also present at presynaptic terminals under physiological conditions, where it transiently associates with SVs [50,51]. In the context of AD pathology, pathogenic tau engages SVs through its N-terminal domain, disrupting presynaptic function—including reducing vesicle mobility and the release rate—and consequently impairing neurotransmission in both Drosophila and rat neurons [15]. Hyperphosphorylated tau is not merely a byproduct of microtubule disassembly; it exhibits novel presynaptic toxic mechanisms [52]. Notably, pathological tau interacts with the SV membrane protein synaptogyrin 3 with high affinity. The genetic deletion of synaptogyrin 3 prevents presynaptic tau from binding to vesicles, alleviates tau-induced defects in vesicle trafficking, and restores neurotransmitter release [53]. These findings strongly support the notion that the tau–SV interaction is a critical driver of AD-related synaptic dysfunction.

The disruption of vesicle trafficking can further compromise the transport and clearance of Aβ and pathological tau. Aβ’s removal and degradation typically occur via proteolytic processing or through vesicular transport to lysosomes, where it is progressively degraded. Delayed vesicular endocytosis increases the delivery of Aβ oligomers to vesicle membranes, potentially facilitating their toxic internalization and consequently impeding Aβ degradation [54]. Among extracellular vesicles, exosomes have been shown to mediate the intercellular transfer of toxic Aβ and hyperphosphorylated tau within the brain, leading to cellular injury and neuronal loss [55,56,57]. Evidence further indicates that targeted inhibition of extracellular vesicle biogenesis and secretion can effectively reduce tau accumulation, ameliorate disease progression in tau transgenic mouse models [58], and simultaneously modulate Aβ clearance, thereby mitigating neurotoxicity [59]. Collectively, these findings underscore the mechanistic link between vesicle trafficking processes and the pathogenesis of AD.

**Figure 2:**
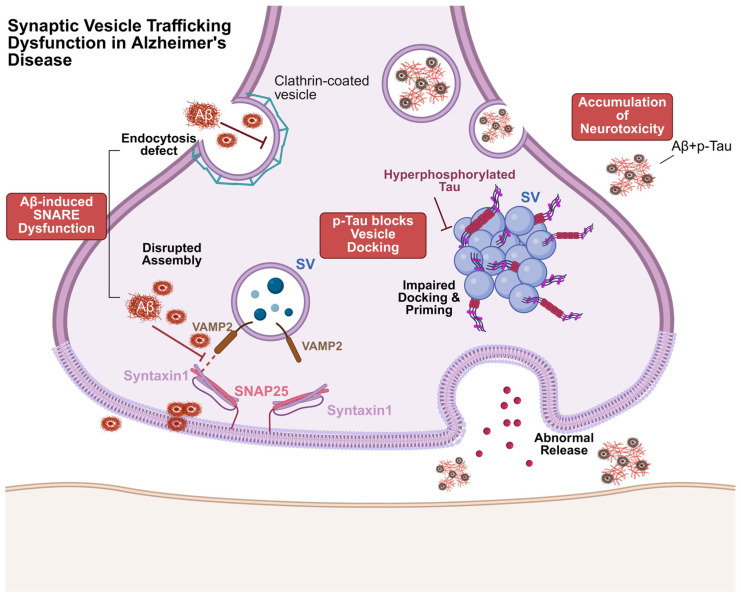
Synergistic disruption of SV trafficking by Aβ and Tau in the presynaptic terminal in AD. Aβ-Induced SNARE Dysfunction: Soluble Aβ oligomers accumulate at the active zone membrane, disrupting the assembly of the SNARE complex. This blockade prevents vesicle fusion (exocytosis) and impairs clathrin-mediated endocytosis (stalled coated pit). p-Tau blocks Vesicle Docking: Cytosolic hyperphosphorylated Tau acts as a physical barrier, abnormally tethering SVs and preventing their docking and priming at the release sites. Accumulation of Neurotoxicity: The synergistic action of Aβ and Tau severs the material foundation of neurotransmission, resulting in an empty synaptic cleft (abnormal release) and the intracellular accumulation of neurotoxic cargo due to failed clearance.

Thus, SV trafficking deficits in AD are not the consequence of a single pathological event (Figure 2). Aβ can disrupt the assembly of SNARE complexes and alter vesicular endo- and exocytosis by modulating the expression of SNARE components and coat-protein-associated factors. Pathological presynaptic tau also interferes with vesicle docking and priming steps. All evidence suggests that Aβ and tau affect the formation of SNARE protein complexes, thereby disrupting the process of SV trafficking, and the synergistic actions of these two pathological agents sever the material foundation of neuronal communication, leading to abnormal neurotransmitter release and further hindering the clearance of pathological cargo, thereby promoting the accumulation of neurotoxins. Nevertheless, there is currently a lack of direct dynamic evidence demonstrating that the presence of Aβ leads to a decrease in the speed and efficiency of SV trafficking. Current studies still fall short of a comprehensive understanding of the downstream effects of these impairments. Future investigations should aim to delineate the intricate interplay between Aβ, tau, and other pathological markers with SV trafficking. Clarifying these interactions will be pivotal for uncovering the precise mechanisms underlying SV dysfunction in AD and ultimately for advancing the development of targeted therapeutic interventions.

### 3.2. Parkinson’s Disease

Parkinson’s disease (PD) has traditionally been characterized by the loss of dopaminergic neurons [60], yet accumulating evidence indicates that presynaptic dysfunction precedes neuronal death by a considerable margin [61]. α-syn, as the central pathogenic protein in PD, exerts significant influence on SV cycling under both physiological and pathological conditions [62].

Under physiological conditions, α-syn is not merely a cytosolic protein but functions as a chaperone that binds to SV-associated proteins. Physiological α-syn helps maintain the orderly arrangement of SVs within the reserve pool, preventing the dispersal of vesicle clusters [63,64]. Evidence shows that α-syn is essential for the assembly of SNARE complexes; it facilitates the pairing of VAMP2 with SNAP 25 and syntaxin 1 at the presynaptic membrane, thereby ensuring efficient neurotransmitter release [65,66]. In lamprey giant reticulospinal synapses, the injection of α-syn antibodies leads to the fragmentation of large vesicle clusters into smaller aggregates or even individual vesicles, demonstrating that α-syn regulates vesicle clustering and docking in vertebrates [16].

In pathological states, excessive or mutant α-syn, such as that encoded by the A53T and A30P variants, undergoes misfolding to form oligomers. Rather than supporting SNARE complex assembly, these oligomers competitively bind to the N-terminal domain of VAMP2, preventing the formation of a stable “zipper” configuration and directly blocking the opening of fusion pores [67,68,69]. Moreover, evidence indicates that one potential toxic mechanism of α-syn oligomers involves their interaction with the v-SNARE protein synaptobrevin 2 (Syb2), and α-syn monomers further potentiate the oligomer-induced inhibition of fusion at submicromolar concentrations, synergistically impairing SNARE-dependent vesicle dynamics, ultimately suppressing dopamine release [70]. Large-scale genome-wide association studies (GWAS) have confirmed that mutations in the *SNCA* gene encoding α-syn are implicated in both familial and sporadic PD. In transgenic mice expressing α-syn or its pathogenic variants, synapses display reduced vesicular exocytosis, ultrastructural SV alterations, decreased SNARE complex abundance, and abnormal synaptic protein levels [71]. Additionally, primary cortical neurons exposed to α-syn for seven days show diminished colocalization of VAMP2 with SNAP 25 [72]. These findings indicate that both altered α-syn expression and conformational changes interfere with SNARE complex formation, thereby inhibiting the membrane fusion of SVs.

Overexpression of α-syn disrupts SV clustering by inhibiting endocytosis, representing another critical pathogenic mechanism [73,74]. Excessive α-syn can impair CME during SV recycling, disrupting the fission and subsequent uncoating of clathrin-coated vesicles. This defect reduces the pool of SVs clustered near the active zone, increases the plasma membrane surface area and the number of endocytic intermediates, and ultimately compromises synaptic function [75]. In PD models, the abnormal accumulation of α-syn leads to excessive vesicle aggregation. This aberrant clustering impairs the ability of vesicles to respond to calcium signals and move into the presynaptic active zone, causing them to accumulate in the peri-active region [74]. The loss of this dynamic mobility interrupts the replenishment of the readily releasable pool, ultimately resulting in synaptic transmission failure during high-frequency stimulation.

In addition to α-syn, the LRRK2-Rab transport network also plays an increasingly important role in the progression of PD [76]. Pathogenic LRRK2 mutations generally increase kinase activity and perturb Rab-dependent membrane trafficking, thereby affecting endocytic recycling, vesicle membrane identity, and lysosomal dynamics [77,78]. Evidence suggests that LRRK2 is not merely a genetic risk factor acting in parallel with α-syn, but rather a central regulator of vesicular trafficking. Hyperactivity of LRRK2 is associated with defects in endocytic transport and impaired vesicle membrane turnover and typically precedes the defects in SV trafficking commonly observed in PD [79]. Compared to the comprehensive regulation of α-syn, LRRK2 appears to exert its effects primarily by disrupting transport processes and disrupting the coordination of membrane compartments [80]. Among the Rab family of proteins, Rab29 and Rab7 also play significant roles in SV trafficking in the pathogenesis of PD [81]. Studies indicate that Rab29 helps localize and activate LRRK2 on Golgi- and endosome-related membranes, thereby facilitating aberrant Rab phosphorylation and downstream trafficking defects in pathogenic settings [82]. By contrast, Rab7 operates primarily on the degradative arm of SV trafficking, coordinating late endosomal maturation and lysosomal delivery [83]. In PD models, Rab7 activation promotes the clearance of α-syn aggregates and reduces toxicity, suggesting that impaired endolysosomal resolution may aggravate presynaptic stress once α-syn accumulates [84,85]. Importantly, these two Rab pathways are not redundant: Rab29 is more closely linked to the initiation and spatial control of LRRK2 signaling, whereas Rab7 is more directly related to the terminal clearance of pathogenic cargo [86].

A further feature that warrants emphasis is the specific changes in the vesicles at the terminals of dopaminergic neurons [87]. Dopamine neurons are not simply the neurons in which key changes occur in PD; rather, they have a wide range of physiological functions, and the vesicular monoamine transporter 2 (VMAT2)-mediated storage of dopamine is a unique feature of their presynaptic biology [88]. Unlike typical glutamatergic SV models, dopaminergic vesicle transport may be regulated by VMAT2, which plays a key role in preventing intracellular dopamine toxicity [89,90]. When vesicular function is impaired, intracellular dopamine homeostasis is disrupted, and oxidative and metabolic processes generate various reactive and toxic byproducts, including reactive oxygen species, DA quinones, and 3,4-dihydroxybenzaldehyde [91]. These toxic byproducts may damage proteins, lipids, and organelles, further leading to the misfolding of α-syn [92]. In this sense, the vesicular trafficking process in dopaminergic neurons serves both neurotransmission and detoxification functions. This may help explain why presynaptic trafficking defects in PD show selective vulnerability in dopaminergic terminals.

**Figure 3:**
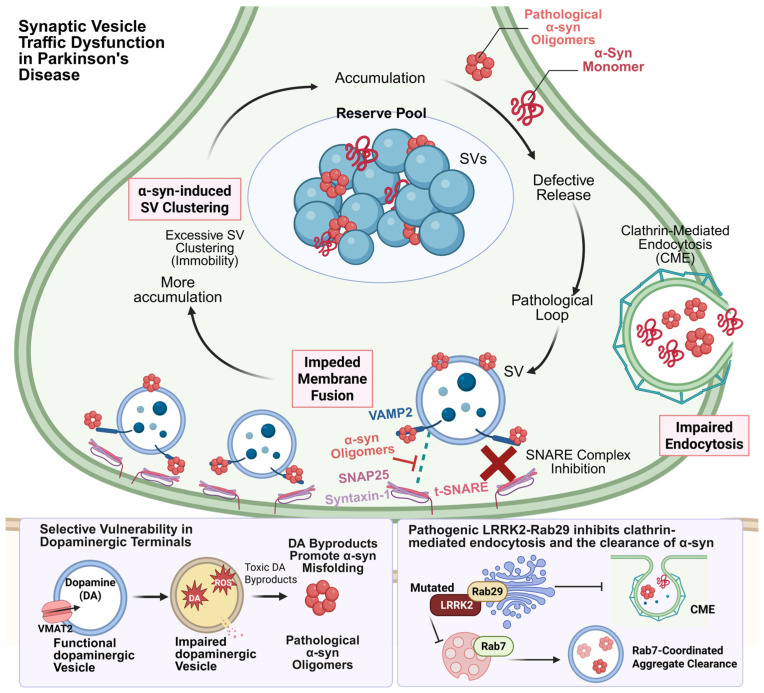
The pathological loop of SV trafficking impairments in PD. α-Syn pathology disrupts vesicle clustering, SNARE-dependent fusion, and clathrin-mediated endocytosis, thereby impairing dopamine release. LRRK2–Rab29-related trafficking defects, reduced Rab7-dependent aggregate clearance, and VMAT2-associated dopamine toxicity further amplify presynaptic stress, forming a vicious cycle that promotes progressive synaptic dysfunction in PD.

Thus, it can be seen that the pathological proteins associated with PD and the SV cycle form a pathological feedback loop in which the two factors are mutually causative. Similarly to AD, in the pathogenesis of PD, the abnormal accumulation of α-syn protein and the accumulation of conformations such as oligomers and preformed fibrils lead to disrupted vesicle aggregation and transport abnormalities [93]. In both diseases, vesicular trafficking dysregulation involves the effects of abnormal presynaptic protein expression on the endocytosis and exocytosis of SVs (Table 1). In contrast, in PD, the α-syn is a key regulator of SV trafficking. Changes in its levels and conformation directly affect the exocytosis and endocytosis of synaptic vesicles, rather than indirectly influencing transport by regulating the expression of SNARE proteins or cytoskeletal proteins. Furthermore, pathological changes in presynaptic vesicles in PD are closely associated with LRRK2-Rab-dependent endocytosis and lysosomal transport dysregulation, as well as specific alterations in dopaminergic neuronal synapses (Figure 3). Consequently, the impairment of SV trafficking in PD may exert a more profound impact on disease pathogenesis, underscoring its importance for elucidating the underlying mechanisms of this disorder. While current evidence supports a link between pathogenic protein changes and SV trafficking defects in the pathogenesis of PD, there is a lack of direct observations of SV trafficking dynamics at the animal or cellular level to demonstrate that SV trafficking defects may directly contribute to the development of PD. The use of super-resolution microscopy techniques (such as STED, STORM, and cryo-electron tomography) to observe SV trafficking may provide a more direct insight into the close relationship between SV trafficking defects and the pathogenesis of PD.

## 4. Intervention Strategies Targeting Presynaptic Mechanisms

SV trafficking has emerged as a promising but still-underdeveloped therapeutic target in neurodegenerative disease. Because SV trafficking is essential for neurotransmitter release, membrane turnover, and synaptic plasticity, its disruption is likely to contribute directly to early synaptic failure in both AD and PD. There are currently no approved therapies specifically designed to repair SV trafficking. Most available strategies act indirectly, by modulating vesicle-associated proteins, improving vesicle recycling, or reducing upstream pathological stress that impairs presynaptic function [106,107]. In this sense, current therapeutic efforts should be viewed as early steps toward synapse-centered intervention rather than as mature vesicle-targeted treatments [108].

Among currently available agents, levetiracetam is one of the most relevant examples because it binds synaptic vesicle protein 2A (SV2A), a key vesicle-associated protein in presynaptic terminals [109]. Although developed as an anti-epileptic drug, levetiracetam has drawn attention in AD because it may reduce abnormal network activity while also influencing synaptic function at the vesicle level [110,111]. Clinical studies in AD-associated hyperexcitability, together with recent experimental data, suggest that SV2A-related modulation may help preserve presynaptic stability under pathological conditions. Even so, levetiracetam is better understood as an indirect synaptic stabilizer rather than as a direct repair strategy for vesicle trafficking itself [112].

In PD, therapeutic interest has increasingly shifted toward pathways that influence vesicle handling upstream. One example is BIIB122 (DNL151), an inhibitor of LRRK2, a kinase closely linked to Rab-dependent membrane trafficking. Because abnormal LRRK2 activity can disturb vesicle recycling and endolysosomal transport, its inhibition may help normalize presynaptic membrane dynamics [113]. Likewise, ambroxol has attracted interest because of its effects on glucocerebrosidase-related lysosomal function, which is closely tied to the turnover of vesicle-associated proteins and toxic cargo such as α-syn [114]. Although neither agent directly targets SNAREs or vesicle fusion machinery, both illustrate an important therapeutic principle: presynaptic vesicle dysfunction may be improved not only by acting on the vesicle cycle itself, but also by correcting the cellular systems that support vesicle recycling and clearance [35].

A central therapeutic challenge is the restoration of vesicle recycling after release [115]. Efficient synaptic transmission depends on rapid endocytosis, membrane uncoating, and reuse of vesicles, and this process is controlled by proteins such as synaptojanin 1, AP2, endophilin, and dynamin [116,117,118]. Genetic and experimental studies strongly support the view that defects in this machinery can drive synaptic dysfunction, particularly in PD [119]. Yet the field has not advanced to the point of having clinically useful drugs that directly restore vesicle recycling. To date, the most realistic strategy is to improve the molecular environment in which recycling occurs—for example, by reducing the α-syn burden, stabilizing endocytic protein interactions, or correcting upstream trafficking imbalances [120]. In this regard, recent evidence that α-syn directly interacts with AP2 further strengthens the idea that endocytic pathways may become tractable therapeutic targets in the future [103].

Overall, the most effective future therapies are unlikely to target a single step of the vesicle cycle in isolation. Instead, they will probably combine disease-specific upstream intervention with direct support of presynaptic resilience (Table 2). In PD, this may involve pairing α-syn- or LRRK2-directed therapy with approaches that improve vesicle recycling or endocytosis [121,122,123]. In AD, it may involve combining a reduction in Aβ/tau-related synaptic stress with strategies that stabilize SV2A-related function or support vesicle turnover under conditions of network hyperactivity [124,125,126]. Progress in this area will also require biomarkers that reflect presynaptic function more directly. Taken together, current evidence suggests that SV trafficking is not yet a mature drug target, but it is increasingly a realistic one. The next stage of research should therefore move from describing vesicle defects to developing interventions that restore vesicle reuse, membrane turnover, and release competence in a measurable and clinically meaningful manner.

## 5. Conclusions

Although a large body of research now indicates that SNARE dysfunction and abnormalities in SV transport are associated with both AD and PD, there remains debate over whether these changes are the primary drivers of these diseases or secondary consequences of neuronal pathology. Research has found that Aβ and α-syn oligomers can directly interfere with the assembly of SNARE complexes involving syntaxin-1A, VAMP2, and SNAP-25, suggesting that certain toxic byproducts of AD and PD may cause dysfunction in SNARE proteins [69,131]. Nevertheless, SNARE protein dysregulation is often accompanied by impaired protein homeostasis, calcium imbalance, mitochondrial dysfunction, and endosomal stress [132,133], all of which further drive disease progression; therefore, it is difficult to attribute it solely to the cause of disease onset or to a secondary manifestation. Therefore, SNARE abnormalities should not be interpreted as uniformly primary lesions; rather, they are likely to function as both early amplifiers and downstream executors of broader presynaptic pathology.

Overall, the precise regulation of SV trafficking relies on a complex set of physiological processes. The synergistic toxicity of Aβ and tau in AD, as well as the aggregation of α-syn, dysregulation of the LRRK2-Rab network, and specific alterations in dopaminergic synapses in PD, all converge at the presynaptic terminal, leading to impaired SV trafficking and disruption of neural transmission (Table 1). Throughout the course of AD and PD, the pathological effects of aberrant protein species and vesicle trafficking defects are bidirectionally linked, each acting as both cause and consequence, thereby forming a vicious cycle that drives disease progression. In the progression of AD and PD, the types of abnormal proteins and the pathological effects of vesicular transport defects are bidirectionally linked; they are both causes and consequences, creating a vicious cycle that drives disease progression.

The key distinction lies in the mechanisms underlying the disruption of SV trafficking: in AD, the exocytic and endocytic phases are primarily affected by the combined action of Aβ and tau; in PD, in addition to the consequences of α-syn overexpression, conformational changes in α-syn directly impede vesicle assembly, impair SNARE complex function, and disrupt endocytosis—highlighting the inherent conformational toxicity in the pathogenesis of PD. Combined with endosomal–lysosomal dysfunction caused by the LRRK-Rab network and specific alterations in dopaminergic synapses, these factors collectively lead to impaired presynaptic transport in PD. These mechanistic differences stem from the etiological heterogeneity of the two diseases, leading to distinct final pathological phenotypes, yet they share the convergent pathological feature of SV transport impairment.

In addition to AD and PD, significantly reduced SV density and exocytosis were observed in striatal neurons of Huntington’s disease model mice [134,135], and the mutated Huntingtin protein (mHTT) disrupts the axonal transport of HTT-Rab 4 vesicles, leading to synaptic and behavioral deficits [136]. Similarly, impaired axonal transport has been observed in Amyotrophic Lateral Sclerosis (ALS) model mice, where the absence of the key gene *C9orf72* leads to a significant reduction in synaptic proteins at excitatory neuron synapses and severe depletion of SVs, while also impairing endocytosis in the cell membrane [137,138,139]. Thus, it can be seen that dysfunction of SV trafficking may occur at various stages during the onset and progression of neurological disorders rather than being a disease-specific phenomenon.

In summary, elucidating the specific mechanisms and molecular targets underlying the dysfunction of SV trafficking that led to neurodegeneration, and restoring these processes before irreversible neuronal damage occurs, holds promise for modulating neural network communication—a strategy that may open new avenues for the development of treatments for neurodegenerative diseases.

## Figures and Tables

**Table 1:** SV Trafficking Dysfunction in AD and PD.

Disease	Pathogenic Protein	Key Impaired Stage	Primary Mechanism of Action	Characteristics of SV Trafficking Dysfunction	Reference
AD	Aβ	Vesicle Clustering and Pool Maintenance	Mitochondrial Trafficking Defect	Impairs mitochondrial transport to synapses, causing local ATP depletion and failure of ATP-dependent vesicle pool maintenance.	[94]
AD	Aβ	Vesicle Clustering and Pool Maintenance	Kinesin Phosphorylation	Induces GSK-3β-mediated phosphorylation of Kinesin light chains, detaching motor proteins and halting vesicle delivery.	[95]
AD	Aβ	Vesicle Clustering and Pool Maintenance	Prion Protein Interaction	High-affinity binding to PrP© disrupts synaptic plasticity and vesicle stability signaling.	[96]
AD	Aβ	Docking, Priming, and the SNARE Assembly	Steric Hindrance of SNARE	Binds directly to the N-terminal domain of Syntaxin-1a, physically blocking the zippering of the SNARE complex.	[48]
AD	Aβ	Calcium-Triggered Fusion	Causes NMDAR/AMPAR dysfunction and Ca^2+^ imbalance	Leads to dysfunction of postsynaptic NMDAR/AMPAR, causing Ca^2+^ overload and temporal disruption; short-term may increase spontaneous release, while long-term results in Ca^2+^-related damage and reduced fusion capacity.	[97]
AD	Aβ	Endocytosis and Recycling	N-cadherin Endocytosis	Delays endocytosis of N-cadherin, indirectly disrupting the structural stability required for vesicle recycling.	[54]
AD	Tau	Vesicle Clustering and Pool Maintenance	“Velcro” Effect	N-terminal domain binds vesicles and crosslinks them into static clusters, restricting their movement to the active zone.	[15]
AD	Tau	Docking, Priming, and the SNARE Assembly	Synaptogyrin-3 Interaction	Binds physically to vesicle protein Synaptogyrin-3, preventing vesicles from entering the Readily Releasable Pool (RRP).	[53]
PD	α-Synuclein	Vesicle Clustering and Pool Maintenance	Liquid–Liquid Phase Separation (LLPS)	Pathological aggregation disrupts the physiological phase separation of Synapsin, sequestering vesicles and preventing mobilization from the reserve pool.	[14]
PD	α-Synuclein	Vesicle Clustering and Pool Maintenance	Membrane Binding and Crosslinking	Crosslinks vesicles via N-terminal membrane binding domains; overexpression leads to excessive clustering and restricts mobility.	[98]
PD	α-Synuclein	Vesicle Clustering and Pool Maintenance	Lipid Raft Disruption	Binds to lipid rafts and alters cholesterol distribution, destabilizing the membrane domains required for vesicle clustering.	[99]
PD	α-Synuclein	Vesicle Clustering and Pool Maintenance	Hsc70 Sequestration	Reduces available Hsc70 chaperone levels, which are necessary for maintaining the proper conformation of vesicle cluster proteins.	[100]
PD	Dopamine vesicle machinery	Vesicle Clustering and Pool Maintenance	SV2C-related vesicular storage defect	SV2C supports vesicular dopamine storage and helps counter dopaminergic toxicity, suggesting a vesicle-specific vulnerability in nigrostriatal terminals.	[88]
PD	Dopamine vesicle machinery	Vesicle Clustering and Pool Maintenance	VMAT2-dependent dopamine sequestration failure	Impaired vesicular loading increases cytosolic dopamine, enhances oxidative stress, and amplifies α-syn-related presynaptic toxicity.	[89]
PD	α-Synuclein	Docking, Priming, and the SNARE Assembly	Chaperone Loss-of-Function	Physiological α-syn promotes SNARE assembly; pathological aggregates lose this function, impairing complex formation.	[11]
PD	α-Synuclein	Docking, Priming, and the SNARE Assembly	Membrane Surface Coating	Oligomers coat the vesicle surface, creating a steric barrier that prevents close contact (docking) with the plasma membrane.	[66]
PD	α-Synuclein	Docking, Priming, and the SNARE Assembly	Directly or indirectly binds to SNARE components	Blocking vesicle docking/priming significantly reduces RRP, leading to a substantial decrease in evoked release and synaptic transmission efficiency.	[101]
PD	α-Synuclein	Calcium-Triggered Fusion	Fusion Pore Constriction	Mutants or oligomers interfere with pore dilation, favoring “kiss-and-run” over full fusion and slowing neurotransmitter release.	[68]
PD	α-Synuclein	Calcium-Triggered Fusion	Co-condensation Hardening	Pathological LLPS with VAMP2 leads to vesicle “hardening,” preventing the membrane fluidity required for fusion.	[65]
PD	α-Synuclein	Calcium-Triggered Fusion	Oligomeric Pores	Annular oligomers permeabilize membranes, causing uncontrolled Ca^2+^ leakage and spontaneous vesicle fusion.	[102]
PD	α-Synuclein	Endocytosis and Recycling	Synaptojanin-1 Inhibition	Reduces Synaptojanin-1 levels/activity, leading to accumulation of PI(4,5)P2 and failure of vesicle uncoating.	[75]
PD	α-Synuclein	Endocytosis and Recycling	Curvature Sensing Defect	Overexpression disrupts the membrane curvature generation required for the initiation of clathrin-mediated endocytosis.	[13]
PD	α-Synuclein	Endocytosis and Recycling	Directly interacts with AP2, regulating its binding to membranes and SV membrane proteins.	Leads to a decrease in the efficiency of clathrin-coated pit formation and SV endocytosis, resulting in reduced SV recycling and ultimately causing a gradual depletion of the functional vesicle pool.	[103]
PD	LRRK2	Endocytosis and Recycling	Hyperactive kinase-dependent SV endocytosis defect	Pathogenic LRRK2, especially G2019S, slows SV endocytosis in ventral midbrain neurons, including dopaminergic neurons, and this defect can be rescued by kinase inhibition.	[79]
PD	LRRK2	Endocytosis and Recycling	Rab-dependent trafficking dysregulation	Increased LRRK2 kinase activity perturbs Rab-regulated membrane trafficking, altering presynaptic vesicle handling and endolysosomal transport.	[80]
PD	Rab29–LRRK2 axis	Endocytosis and Recycling	Membrane recruitment and activation of LRRK2	Rab29 recruits LRRK2 to membrane organelles and stimulates its kinase activity, thereby acting upstream of trafficking defects rather than as a simple downstream effector.	[104,105]
PD	Rab7	Endocytosis and Recycling	Late endosomal–lysosomal clearance of α-syn	Rab7 promotes autolysosomal degradation of α-syn aggregates, reduces α-syn toxicity, and links presynaptic dysfunction to impaired degradative trafficking.	[85]

**Table 2:** Representative drugs or methods related to SV trafficking in AD and PD.

Therapeutic Focus	Representative Therapeutic Drugs/Methods	Disease	Proposed Relevance to SV Trafficking	Reference
SV2A/vesicle-associated synaptic stabilization	Levetiracetam	AD	Binds SV2A and may stabilize vesicle-associated synaptic function; also reduces network hyperexcitability linked to presynaptic stress	[112]
SV2A/vesicle-associated synaptic stabilization	Brivaracetam	Potentially AD/PD-related	Higher-affinity SV2A ligand than levetiracetam; supports the druggability of vesicle protein SV2A, but direct AD/PD evidence remains limited	[127]
LRRK2-Rab trafficking regulation	BIIB122 (DNL151)	PD	Targets LRRK2, an upstream regulator of Rab-dependent membrane trafficking; may improve vesicle recycling and endolysosomal transport	[113]
Lysosomal support linked to vesicle turnover	Ambroxol	PD	Improves glucocerebrosidase-related lysosomal function, which may support turnover of vesicle-associated proteins and α-syn cargo	[114]
α-Syn burden reduction upstream of vesicle recycling defects	Prasinezumab	PD	Does not directly target SV machinery, but may reduce α-syn-related presynaptic stress that impairs docking, fusion, and recycling	[128]
Dopamine vesicle loading/VMAT2-dependent vesicle protection	Gastrodin	PD	Upregulates VMAT2 through MEK-dependent signaling, helping maintain dopamine vesicle storage and reduce cytosolic dopamine toxicity	[90]
Lysosome–vesicle interface/α-syn clearance	Acidic nanoparticles	PD	Restore lysosomal function and may indirectly improve vesicle turnover in α-syn-driven synaptic pathology	[121]
Extracellular vesicle trafficking/pathogenic cargo removal	Engineered hybrid exosomes as Aβ nano scavengers	AD	Multi-target extracellular vesicle-based strategy that may improve pathological cargo handling linked to vesicle trafficking	[59]
Extracellular vesicle secretion/tau propagation pathway	P2RX7-related extracellular vesicle modulation	AD	Affects tau and mitochondrial loading into extracellular vesicles; relevant to pathological vesicle-mediated spread rather than classical synaptic SV cycling	[58]
Clathrin-mediated endocytosis kinase	AAK1 inhibitors	Potential AD/PD relevance	Support the druggability of endocytosis pathways that control vesicle internalization	[129]
Direct fusion machinery modulation	SNARE-modulating peptides/SNARE mimetics	Conceptual for AD/PD	Experimental tools that can modify SNARE-binding interfaces and directly modulate vesicle docking/fusion	[130]

## Data Availability

No new data were created or analyzed in this study. Data sharing is not applicable to this article.

## Abbreviations

The following abbreviations are used in this manuscript:

SV	Synaptic vesicle
AD	Alzheimer’s disease
PD	Parkinson’s disease
APP	Amyloid precursor protein
Aβ	β-amyloid
α-Syn	α-synuclein
RRP	Readily releasable pool
STXBP1	Syntaxin-binding protein 1
SNAREs	Soluble N-ethylmaleimide-sensitive factor attachment protein receptors
VAMP2	Vesicle-associated membrane protein 2
SNAP25	25 kDa synaptosomal-associated protein
STX-1	Syntaxin-1
CaMKIV	Calmodulin kinase IV
DNM	Dynamin.
